# Synthesis, DFT and molecular docking of novel (*Z*)-4-bromo-*N*-(4-butyl-3 (quinolin-3-yl)thiazol-2(3*H*)-ylidene)benzamide as elastase inhibitor

**DOI:** 10.1186/s13065-023-00985-4

**Published:** 2023-08-07

**Authors:** Muhammad Naeem Mustafa, Pervaiz Ali Channar, Syeda Abida Ejaz, Saira Afzal, Mubashir Aziz, Tahira Shamim, Aamer Saeed, Aisha A. Alsfouk, Rabail Ujan, Qamar Abbas, Tuncer Hökelek

**Affiliations:** 1 Department of Chemistry, Quaid-I-Azam University, Islamabad, 45320 Pakistan; 2 Department of Basic Sciences and Humanities, Faculty of Information Sciences and Humanities, Dawood University of Engineering and Technology Karachi, Karachi, 74800 Pakistan; 3 Department of Pharmaceutical Chemistry, Faculty of Pharmacy, The Islamia University of Bahawalpur, Bahawalpur, 63100 Pakistan; 4 Faculty of Pharmacy, The University of Lahore, Lahore, Pakistan; 5 University College of Conventional Medicine, Faculty of Medicine and Allied Health Sciences, The Islamia University of Bahawalpur, Bahawalpur, 63100 Pakistan; 6 Department of Pharmaceutical Sciences, College of Pharmacy, Princess Nourah bint Abdulrahman University, P.O Box 84428, Riyadh, 11671 Saudi Arabia; 7 Dr. M. A. Kazi Institute of Chemistry, University of Sindh, Jamshoro, Pakistan; 8 Department of Biology, College of Science, University of Bahrain, Sakhir Campus, Sakhir, 32038 Bahrain; 9 College of Natural Sciences, Department of Biological Sciences, Kongju National University, Gongju, 32588 Republic of Korea; 10 Department of Physics, Faculty of Engineering, Hacettepe University, Beytepe-Ankara, Ankara, 06800 Turkey

**Keywords:** Elastase, Molecular docking, Hirshfeld, DFT studies, Crystallography

## Abstract

A new compound, C_23_H_20_BrN_3_OS, containing a quinoline-based iminothiazoline with a thiazoline ring, was synthesized and its crystal and molecular structures were analyzed through single crystal X-ray analysis. The compound belongs to the triclinic system *P *− 1 space group, with dimensions of a = 9.2304 (6) Å, b = 11.1780 (8) Å, c = 11.3006 (6) Å, α = 107.146 (5)°, β = 93.701 (5)°, γ = 110.435 (6)°, Z = 2 and V = 1025.61 (12) Å^3^. The crystal structure showed that C–H···N and C–H···O hydrogen bond linkages, forming infinite double chains along the b-axis direction, and enclosing R_2_^2^(14) and R_2_^2^(16) ring motifs. The Hirshfeld surface analysis revealed that H…H (44.1%) and H…C/C…H (15.3%) interactions made the most significant contribution. The newly synthesized (Z)-4-bromo-N-(4-butyl-3 (quinolin-3-yl)thiazol-2(3H)-ylidene)benzamide, in comparison to oleanolic acid, exhibited more strong potential against elastase with an inhibition value of 1.21 µM. Additionally, the derivative was evaluated using molecular docking and molecular dynamics simulation studies, which showed that the quinoline based iminothiazoline derivative has the potential to be a novel inhibitor of elastase enzyme. Both theoretical and experimental findings suggested that this compound could have a number of biological activities.

**Supplementary Information:**

The online version contains supplementary material available at 10.1186/s13065-023-00985-4.

## Introduction

**Fig. 1:**
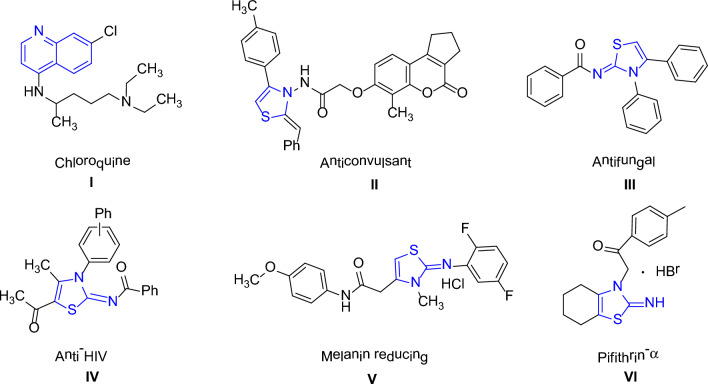
Illustration of biologically significant thiazoline and quinoline derivatives [19–23]

The presence of a quinoline moiety (as illustrated in Fig. 1) in different type of scaffolds is responsible for the variety of the biological activities, including anthelmintic, analgesic, anticonvulsant, antibacterial, cardiotonic, anti-inflammatory, antimalarial, and antifungal activities [1]. Many pharmacologically active compounds and natural products contain quinoline functionality, including Cinchona Alkaloids [2]. Chloroquine I, a well-known quinoline-based medicine, has played a crucial role in eradicating and controlling malaria for several decades. This drug has been demonstrated to impact the parasite's life cycle during the blood stages [3].

Another group of compounds i.e., Thiazoline-based heterocyclic compounds have been shown to possess various pharmacological applications in the medicinal chemistry as well as in industry including their antipyretic, antiallergic [4], antibiotic [5], anticonvulsant II [6, 7], antifungal III [8], antihypertensive [9], anti-HIV IV [10], anti-inflammatory [11], antirheumatic, antitumor [12], antimalarial, analgesic [13], and cytotoxic effects [14]. Among them, 3-methylthiazolidine is a potent inhibitor of indole ethylamine N-methyltransferase (INMT) [15] and has potential as a therapeutic agent for treating schizophrenia [16]. Thiacloprid, a commercially available iminothiazoline insecticide developed by Bayer CropScience [17], and 2-imino-3-(benzoylmethyl)thiazolidine, which has effective radioprotective activity against γ-radiation [18], are other examples of thiazoline-based compounds with various applications. Hydroxy thiazole carboxylate II is well-known potential inhibitor of IF-α prolyl hydroxylase, while PS-028 is a known antagonist, which selectively inhibit GPIIb/IIIa [19, 20].

The scaffold V containing 2-Imino-1,3-thiazoline has exhibited significant dose-dependent inhibition of melanin production, indicating its potential as a skin whitening agent [21]. Pifithrin-α VI, an iminothiazoline compound, is known for its ability to reversibly inhibit p53-dependent gene transcription and p53-mediated apoptosis, making it a valuable therapeutic agent [22]. In addition, the use of thiazolines as plant growth regulators has shown potential for enhancing crop growth and yield, improving stress tolerance, and reducing environmental impacts associated with conventional farming practices [23]. As such, thiazolines are poised to play an increasingly important role in the future of agriculture as a key component of integrated pest management and sustainable crop production. Additionally, natural products such as mirabazoles, tantazoles, and thiangazole, which contain thiazoline functionality, have demonstrated anticancer and anti-HIV activities [14].

The skin is a primary target of oxidative stress because reactive oxygen species (ROS) can both be produced by and be found in the skin itself. ROS are produced as a natural byproduct of regular metabolic processes; they are an essential component of normal cellular function; nevertheless, they rarely cause significant damage due to the presence of intracellular mechanisms that mitigate the effects of their toxicity [24]. Antioxidants reduce the harmful effects of reactive oxygen species (ROS) and have the ability to impede or even revert the progression of many of the events that lead to epidermal toxicity and disease [25]. Prolonged exposure to ultraviolet radiations causes the stimulation of reactive oxygen species (ROS) in the skin which results in premature aging [26]. The storage of ROS in the skin can play a role for the activation of disease-causing enzymes including elastase and tyrosinase [27]. Elastase is a serine protease enzyme that cleaves elastin, a component of the extracellular matrix in connective tissue help in manage the elasticity of skin through the formation of elastic fibers in the skin. Several studies suggest that skin aging effect is associated with decomposition of elastin [28]. The active site of elastase consists of the catalytic triad of residues: His57, Asp102, and Ser195, with His57 acting as a proton acceptor, Aspartic as a proton donor, and Serine as the nucleophile that attacks the substrate peptide bond. Elastase also has an oxyanion hole that stabilizes the transition state during catalysis [29]. Other important residues for inhibition include Gly216 and Ala218, which form the "oxyanion hole" that stabilizes the tetrahedral intermediate during catalysis, and Valine and Serine residues, which form a hydrophobic pocket that accommodates the substrate. The structure of elastase also includes a flexible loop region, residues 192–205, which undergoes conformational changes during catalysis and can affect inhibitor binding. Overall, the availability of structural data and knowledge of the active site residues of elastase are essential for the design of effective inhibitors for this enzyme [30].

A potential elastase inhibitor with good antioxidant potential was explored using quinoline-based iminothiazoline analogue. Experimental results were validated by computational approaches, including density functional theory calculation, molecular docking, and molecular dynamics studies. The study suggested that the designed molecule has potential for further exploration at the molecular level, leading to the synthesis of drug-like molecules with greater potential and safety profiles. This approach may provide an efficient way to protect against skin aging by inhibiting elastase enzyme activity.

## Results and discussion

### Synthesis

**Scheme 1:**
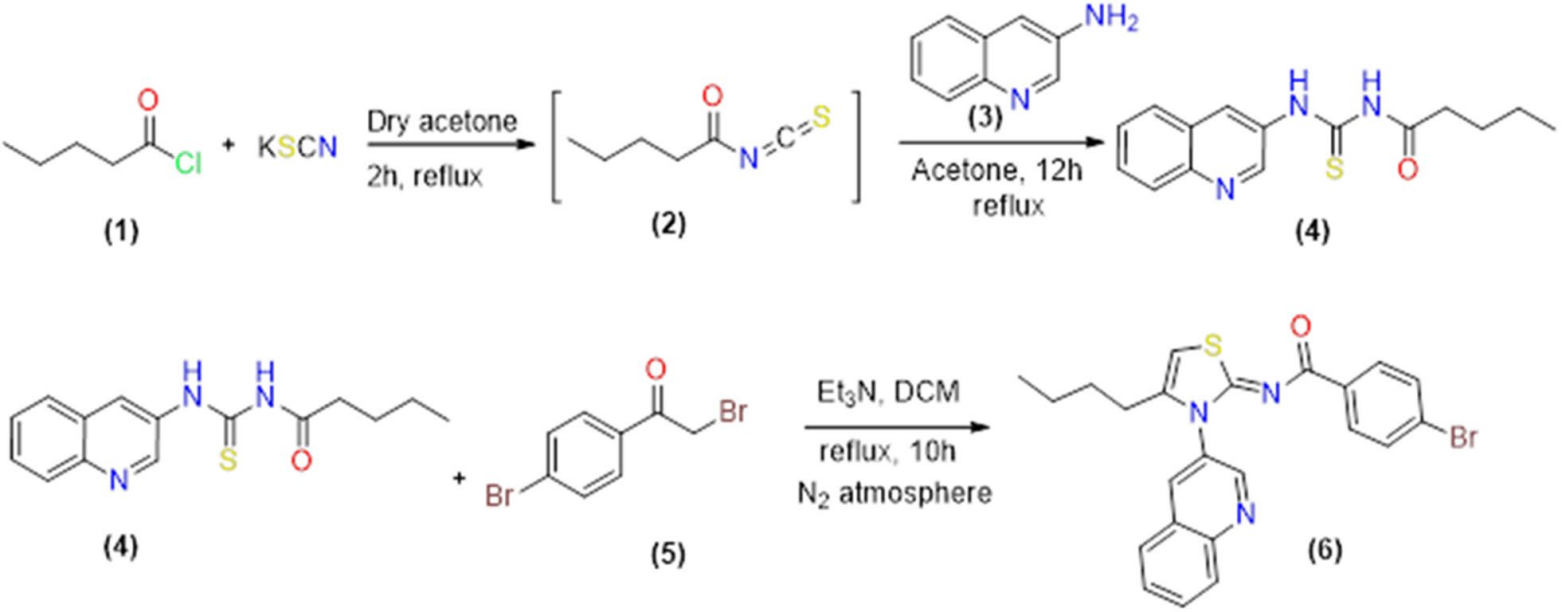
Synthesis scheme for quinolinyl iminothiazoline 6

A novel iminothiazoline analogue with a quinoline base was synthesized using a multi-step chemical process. The initial reaction involved the combination of pentanoyl chloride 1 with potassium thiocyanate in a dry acetone solvent, followed by the addition of 3-aminoquinoline 3 to generate acyl thiourea 4. The purified acyl thiourea 4 was then mixed with p-bromophenacylbromide 5 to produce (Z)-4-bromo-N-(4-butyl-3-(quinolin-3-yl)thiazol-2(3H)-ylidene)benzamide 6, as demonstrated in Scheme 1. The use of dry solvents throughout the process helped to prevent the hydrolysis of isothiocyanate 2 due to moisture. Moreover, the final step was performed under a nitrogen atmosphere to prevent any unwanted side products from forming.

### Mechanism

**Scheme 2:**
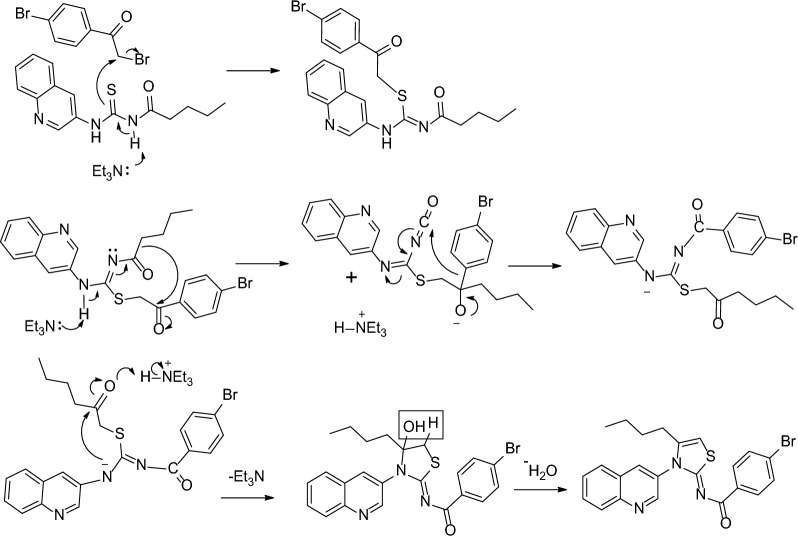
Mechanistic route for conversion of acyl thiourea to quinolinyl iminothiazoline 6

Scheme 2 shows the mechanism of the synthesis, which proceeds through several steps. Initially, sulfur attacks the alpha carbon of *p*-bromophenacylbromide, and the acidic N-1 proton transfers to a base to form the isothiourea intermediate 7. This intermediate undergoes rearrangement of alkyl and aryl groups, facilitated by the removal of a proton from N-3. The rearranged intermediate 8 has a negative charge on N-3, which then attacks the carbonyl group. The final step involves dehydration, resulting in the formation of the product. The spectral characterization of the compound is given in the Additional file 1: Table S1.

### X-ray data collection, structure solution, and refinement

**Fig. 2:**
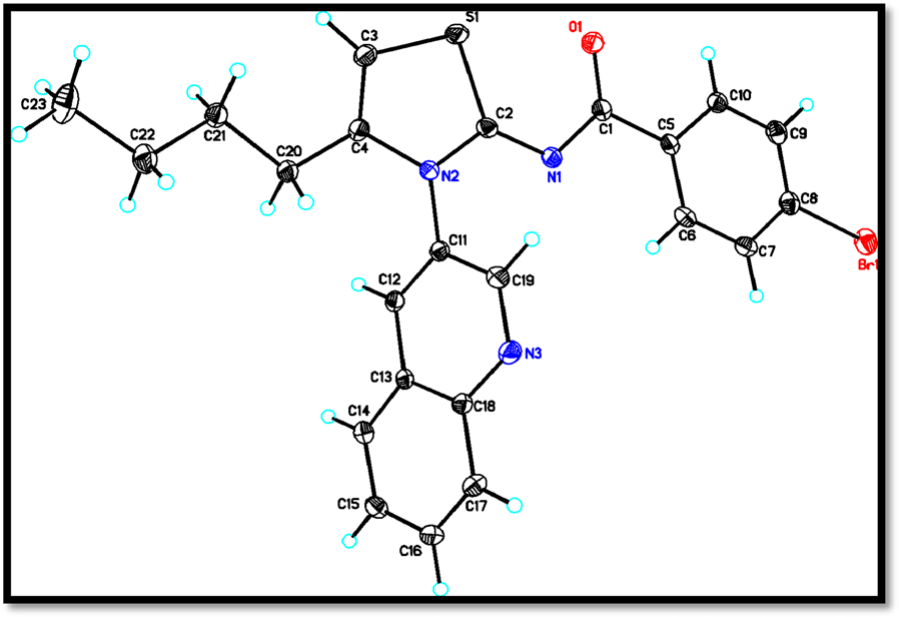
X-ray structure analysis of quinolinyl iminothiazoline 6

The x-ray analysis revealed that the analyzed compound contains a bicyclic system consisting thiazole ring. The thiazole ring is not visible in the molecular structure due to its conformation, while the pyridine ring forms a part of the bicyclic system. The molecular structure, depicted in Fig. 2, shows that planar rings A (S1/N2/C2–C4), B (C5–C10), C (N3/C11–C13/C18/C19), and D (C13–C18) are positioned at dihedral angles of A/B = 6.03(6)°, A/C = 66.27(6)°, A/D = 67.56(6)°, B/C = 72.01(5)°, B/D = 73.34(6)°, and C/D = 1.96(5)°. Additionally, atoms Br1, C1, and O1 are situated − 0.0598(3) Å, − 0.0325(20) Å, and 0.0527(15) Å away from the best least-square plane of ring B, respectively, indicating that they are nearly coplanar with the ring plane.

**Fig. 3:**
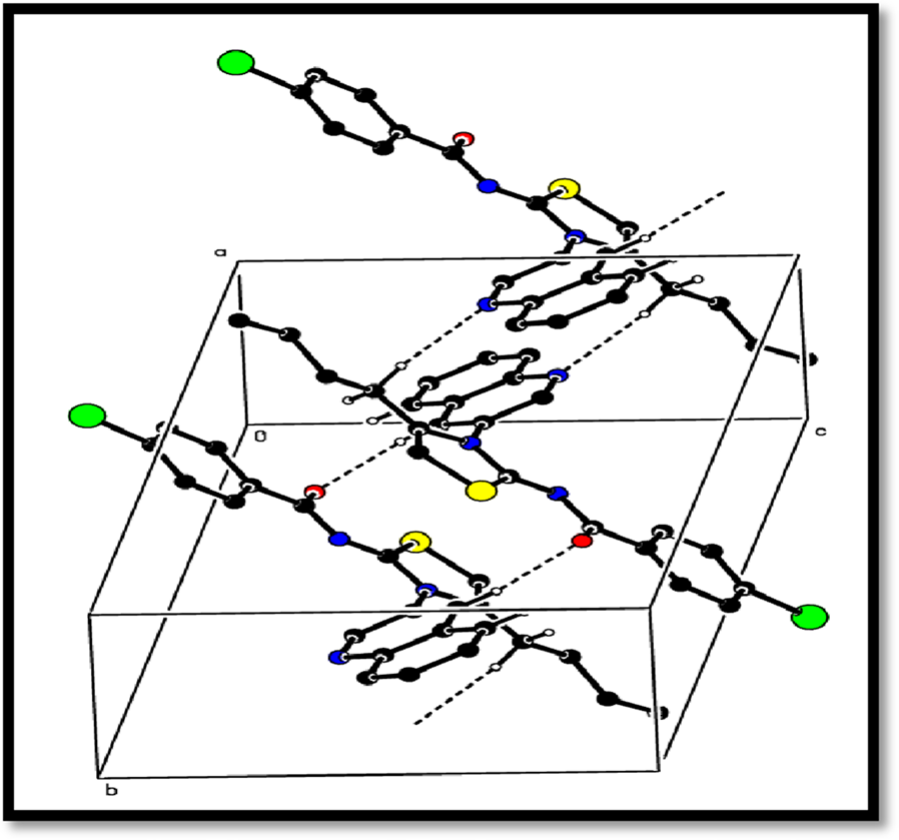
A partial packing diagram viewed down the b-axis direction. Intermolecular C–H···O and C–H···N hydrogen bonds represented as dashed lines

The crystal structure analysis revealed that the molecules in the compound form infinite double chains along the b-axis direction through C–H···N and C-H···O hydrogen bonds. These chains form R_2_^2^(14) and R_2_^2^(16) ring motifs (shown in Fig. 3). Additionally, π–π interactions were observed with centroid to centroid distances of Cg2…Cg2^i^ = 3.7364(13) Å and Cg3…Cg3^ii^ = 3.4714(12) Å [symmetry codes: (i) 1 − x, 1 − y, 2 − z, (ii) − x, − y, 1 − z, where Cg2 and Cg3 are the centroids of the rings B and C, respectively], and a weak C–H···π interaction (Table 1) further reinforce a three-dimensional architecture (Fig. 3).

**Table 1:** The representation of hydrogen-bond geometry (Å, º) of the synthesized Compound

*D*–H···*A*	*D*–H	H···*A*	*D*···*A*	*D*–H···*A*
C20–H20*A*···N3^ii^	0.99	2.53	3.489 (3)	163
C21–H21*B*···Cg2^iii^	0.99	2.90	3.760(2)	149
C12–H12···O1^i^	0.95	2.29	3.227 (2)	171

Codes for symmetrical arrangements include (i) x + 1, y + 1, z + 1; (ii) x + 1, y + 2, z + 1; and (iii) 1 − x, 1 − y, 1 − z > . Cg2 is the center of attention in ring B (C5–C10)

The distance between atoms involved in hydrogen bonding in the titled compound was calculated and three types of hydrogen bonds were observed: 3 D----H and H---A. More information about the hydrogen bonding can be found in Table 1.

Intermolecular C–H···O and C–H···N hydrogen bonds were linking the titled molecule. The compound was crystallized in triclinic system according to X-ray diffraction data. The dimensions and volume of the unit cell were as follows; a = 9.2304 (6) Å, b = 11.1780 (8) Å, c = 11.3006 (6) Å and V = 1025.61 (12) (Å^3^).

### Hirshfeld surface analysis

**Fig. 4:**
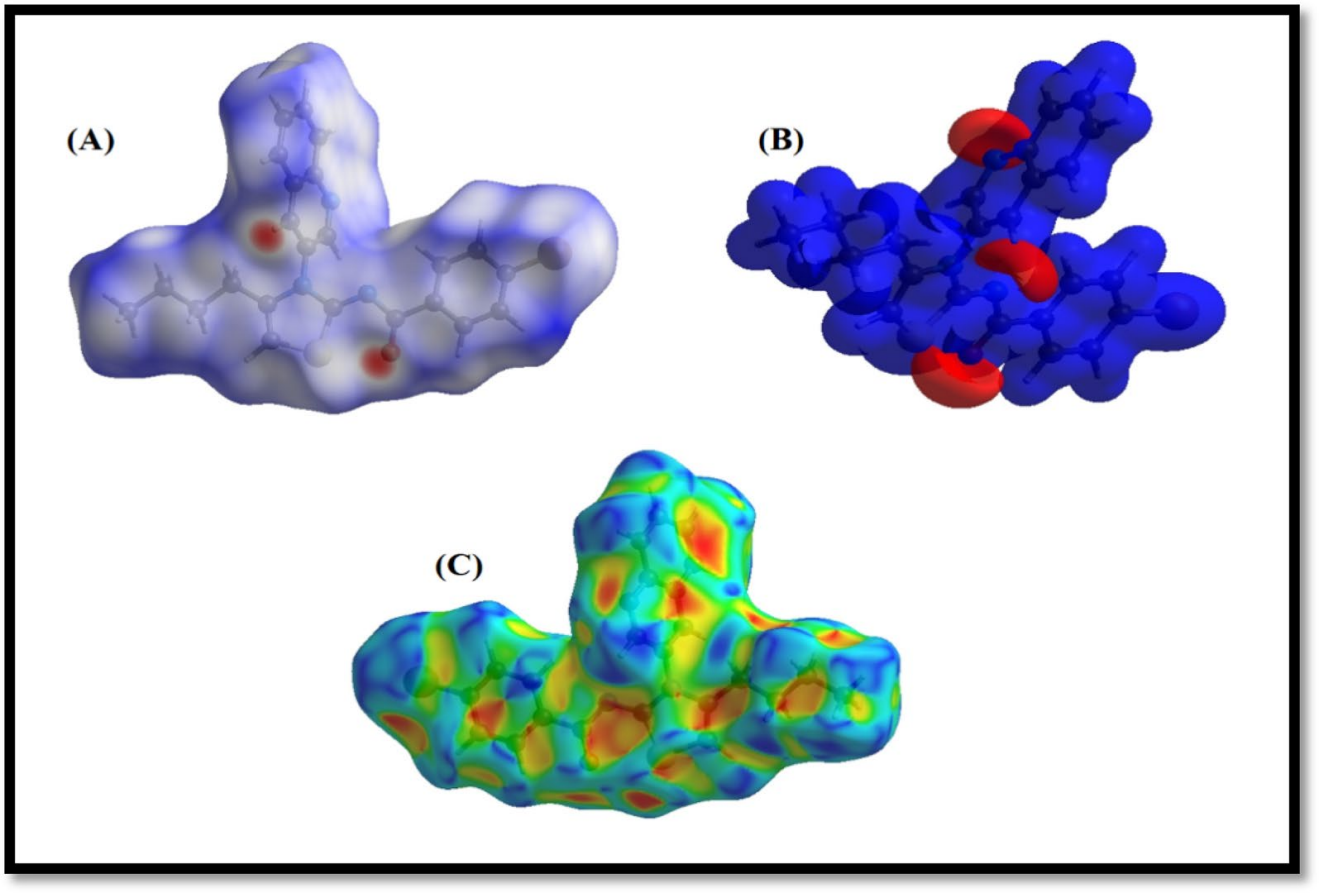
It displays three different 3D views of the compound. The first view (**A**) shows the compound plotted over d norm, while the second view (**B**) displays the compound V/S electrostatic potential energy. Finally, the third view (**C**) shows the compound V/S shape-index

**Fig. 5:**
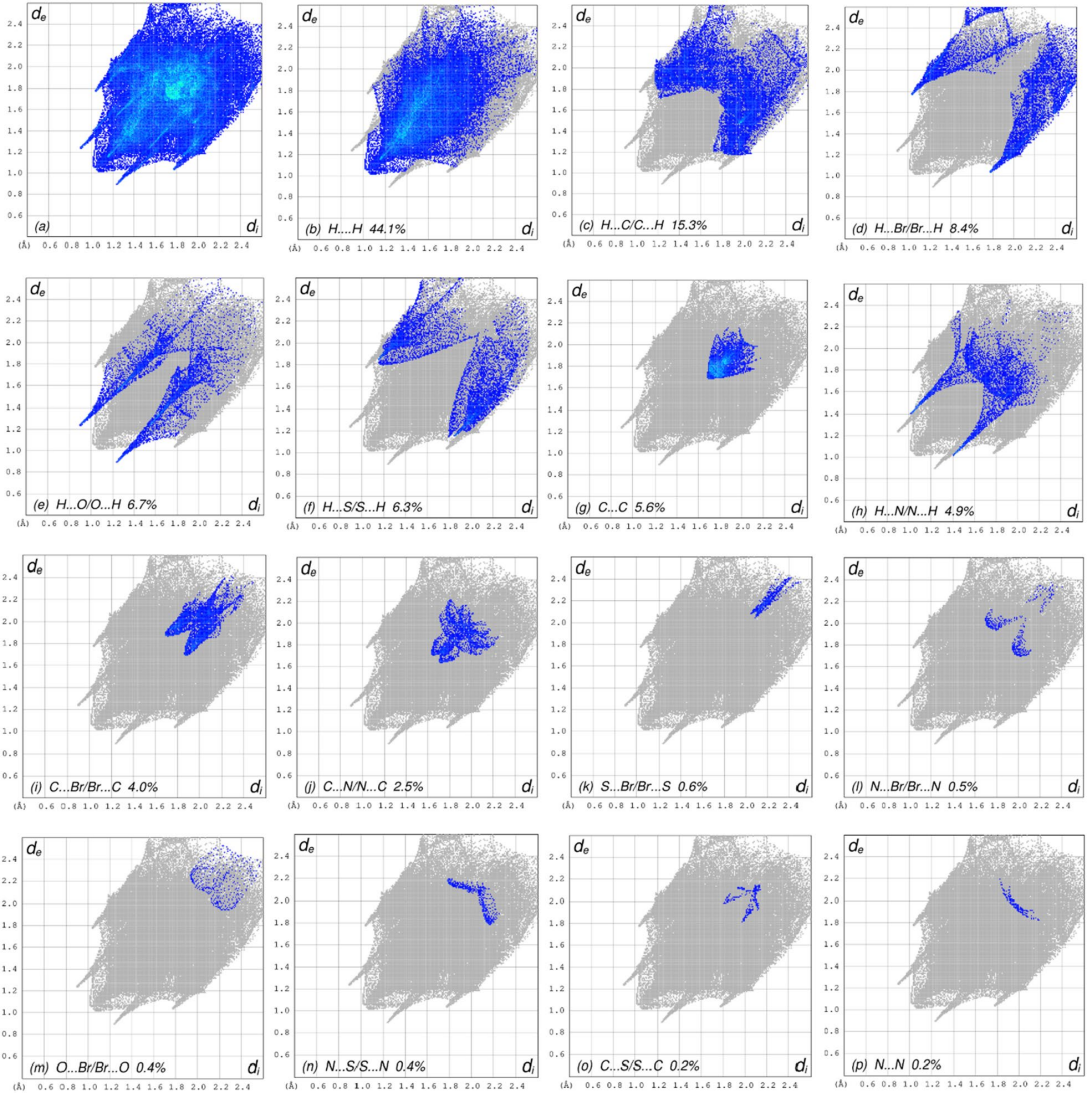
The comprehensive 2D fingerprint plots displaying all of the interactions involving compound 6 (**a**), which are separated into (**b**) H … H(44.1%), (**c**) H … C/C … H (15.3%), (**d**) H … Br/Br … H (8.4%), (**e**) H … O/O … H (6.7%), (**f**) H … S/S … H (6.3%), (**g**) C … C (5.6%), (**h**) H … N/N … H (4.9%), (**i**) C … Br/Br … C (4.0%), (**j**) C … N/N … C (2.5%), (**k**) S … Br/Br … S (0.6%), (**l**) N … Br/Br … N (0.5%), (**m**) O … Br/Br … O (0.4%), (**n**) N … S/S … N (0.4%), (**o**) C …S/S … C (0.2%) and (**p**) N … N (0.2%)

Contact distances and different electrostatic potential were expressed as different-coloured regions of Hirshfeld surface analysis. Red-colour regions was representing shorter contacts whereas blue-colour regions exhibited longer contacts. Various electrostatic regions are represented in Figs. 4 and 5.

Figure 5 provides an illustration of the 2D fingerprint plot of the compound. This plot depicts the numerous interactions, such as H…H, H…C/C…H, H…Br/Br…H, H…O/O…H, H…S/S…H, C…C, H…N/N…H, C…Br/Br…C, C…N/N…C, S…Br/Br…S, N…Br/Br…N, O…Br/Br…O, N…S/S…N, C…S/S…C and N…N [31] are separated and shown in Fig. 5b–p. The contribution of the most significant interaction, H…H interaction, to the crystal packing is 44.1%, as shown in Fig. 5b with d_e_ = d_i_ = 1.05 Å. The detailed explanation can be found in the Additional file 1.

**Fig. 6:**
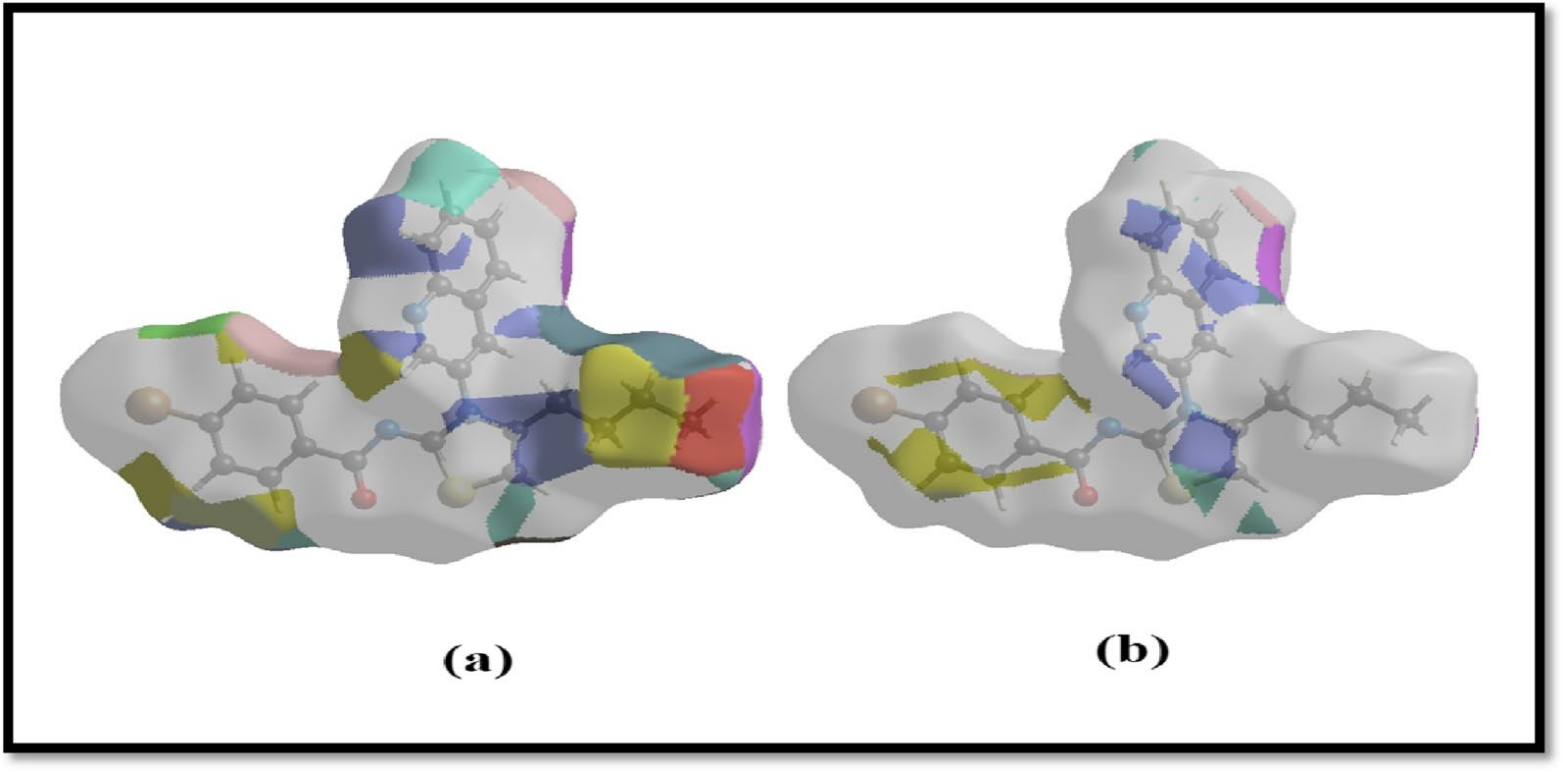
The Hirshfeld surface illustration; (**a**) H … H (**b**) H … C/C … H interactions

HS representation of hydrogen bonds was illustrated using D_norm_ function. Figure 6a is representing different colored surface for H…H interactions. The red color surface is depicting strong dominance of H…H interactions whereas blue colored region is representing slightly weaker interactions. In contrast, Fig. 6b is illustration of H … C/C … H interactions. These interactions were slightly compromised and localized to certain areas of titled compound. Detailed representation is provided below in Fig. 6.

### Crystal voids

**Fig. 7:**
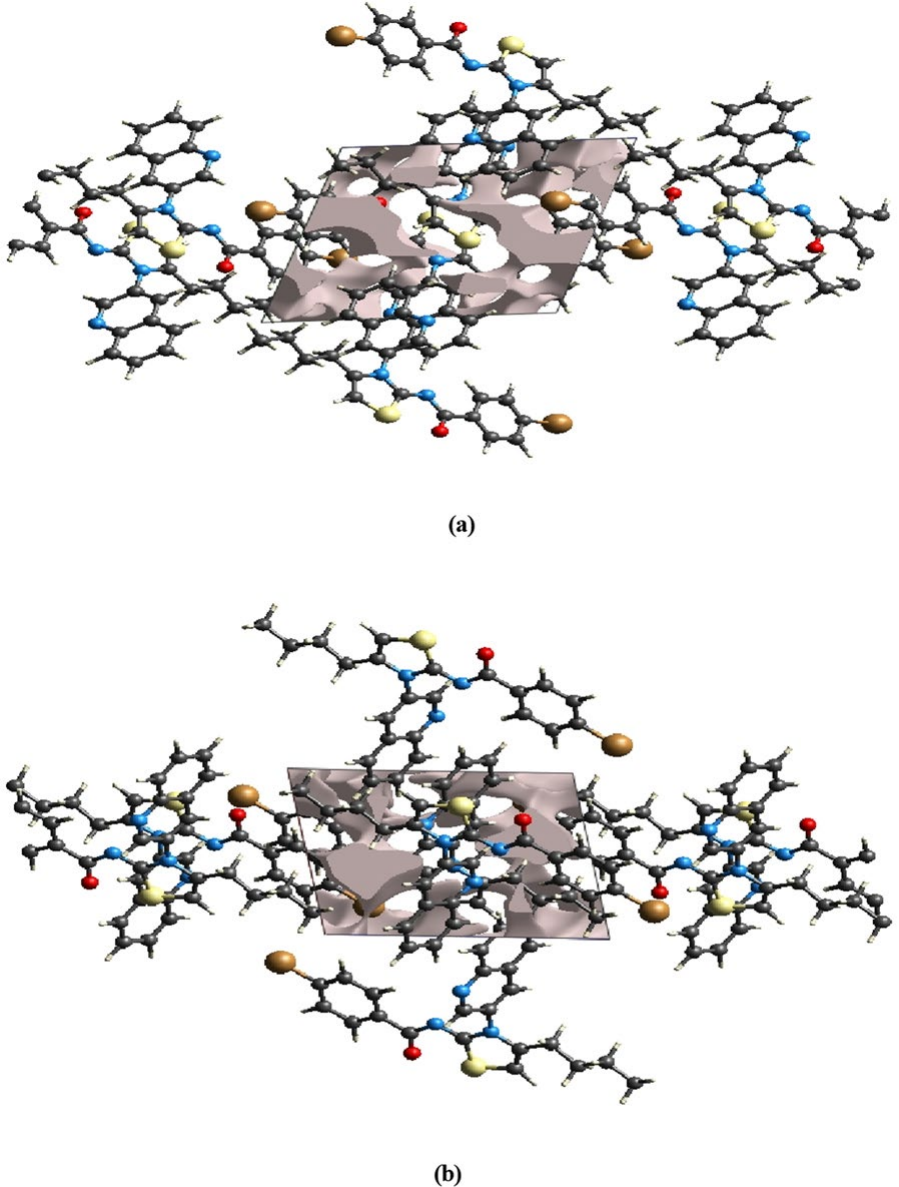
Graphical representation of crystal voids of the title compound (**a**) along a-axis and (**b**) along b-axis

The mechanical stability of a crystal is largely determined by the strength of its packing, as applied mechanical forces depend on how tightly molecules are packed. A crystal with large empty spaces or voids is more susceptible to external forces, making it less mechanically stable. In order to assess the mechanical stability of the crystal under investigation, we performed crystal void analysis by adding electron densities in the asymmetric units. The crystal void volume was measured to be 112.74 Å^3^, while the free spaces percentage in the unit cell was found to be 10.99%. This indicates that there are no large cavities in the crystal packing, suggesting that the crystal has good mechanical stability. [32, 33]. These results are further supported by the optimized crystal structure shown in Fig. 7a, b. The information on voids in the crystal structure of a ligand can provide insights into the binding mode and dynamics of the ligand with its target protein. The presence of large voids or cavities in the crystal structure of the ligand may indicate that the ligand has flexible regions or conformations that can potentially interact with different regions of the protein surface. This can have an effect on the docking score, as well as the expected binding mechanism of the ligand to the protein, as well as the stability and dynamics of the system when it is simulated [33].

On the other hand, if the crystal structure of the ligand reveals that it has few voids or contacts that are tightly packed, this may indicate that the structure is more solid or stable and that it interacts with particular parts of the protein surface. During the simulation investigations, this can provide more confidence in the projected binding mechanism and stability of the ligand. Because of this, the information on voids and packing in the crystal structure of a ligand can be a helpful complement to docking and dynamics studies. This is because it can help to understand the potential binding modes and conformations of the ligand with the protein surface, and it can also help to optimize the stability and efficacy of the ligand as a drug candidate [33, 34].

### Density functional theory (DFTs) studies

**Fig. 8:**
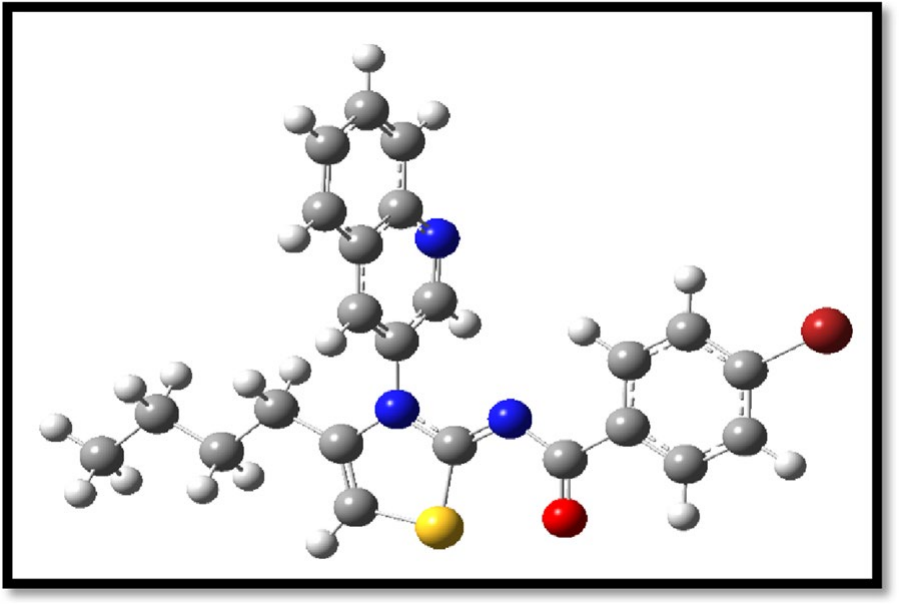
Optimized structure

The molecule that was synthesized was then optimized with regard to its structural geometries, and frequency calculations were carried out with the assistance of the DFT/B3LYP functional correlation and the cc-pVDZ basis set. The most important purpose of optimization was to rearrange the atoms in the molecule in such a way that it would result in the lowest possible amount of energy being used. In addition to that, frequency calculations were performed for each individual atom that was included in the manufactured molecule. Figure 8 is a depiction of the optimized geometrical properties of the newly manufactured molecule.

The optimization method revealed no imaginary frequencies, indicating that the present geometries were true local minima. Table 2 summarizes the optimized geometry parameters, including optimization energy and polarizability.

**Table 2:** Optimized geometry parameters

Sr. no	Compound Code	Optimization energy (Hartree)	Polarizability (α) (a.u.)
01	**6**	-4053.730138	212.856000

The frontier molecular orbitals, or FMOs, are a type of molecular orbital that can provide light on a molecule's reactivity as well as its chemical properties, such as its hardness and softness. In the current effort of creating a ligand for the inhibition of elastase, the study of FMOs can be helpful in understanding the potential interactions that could occur between the ligand and the active site of the enzyme, as well as in optimizing the chemical characteristics of the ligand for improved binding affinity and inhibition potency. The nucleophilic character of a molecule is often connected with the HOMO, while the electrophilic character is generally linked with the LUMO. The amount of energy that differs between these orbitals is referred to as the HOMO–LUMO gap, and it is a crucial predictor of both the reactive and stable properties of a molecule. The HOMO and LUMO energies were determined to be − 0.2165 and − 0.075 eV, respectively, in the course of this research. As was said in the previous section [35], the comparatively modest energy gap of 0.1430 eV shows that compound 6 possesses an adequate amount of reactivity. The energy gap can be thought of as a measurement of the amount of energy needed to excite an electron from the most highly occupied molecular orbital (HOMO) to the least highly occupied molecular orbital (LUMO). A small energy gap implies that the compound can easily undergo electronic transitions and can react with other molecules or undergo photochemical reactions. Therefore, the small energy gap of compound 6 suggests that it is more reactive and can readily undergo chemical reactions [35, 36]. Moreover, the compound's chemical hardness and softness values further support its reactivity. Notably, compound 6 displayed a softness value of 6.99, indicating that it is highly polarizable and thus more likely to undergo reactions. The electronic parameters of the synthesized crystal are presented in Table 3.

**Table 3:** Electronic Parameters of selected crystal (6)

Compound	E_HOMO_ (eV)	E_LUMO_ (eV)	∆E_gap_ (eV)	Hardness (η)	Softness (S)
6	− 0.12197	0.02102	0.1430	0.071	6.99

**Fig. 9:**
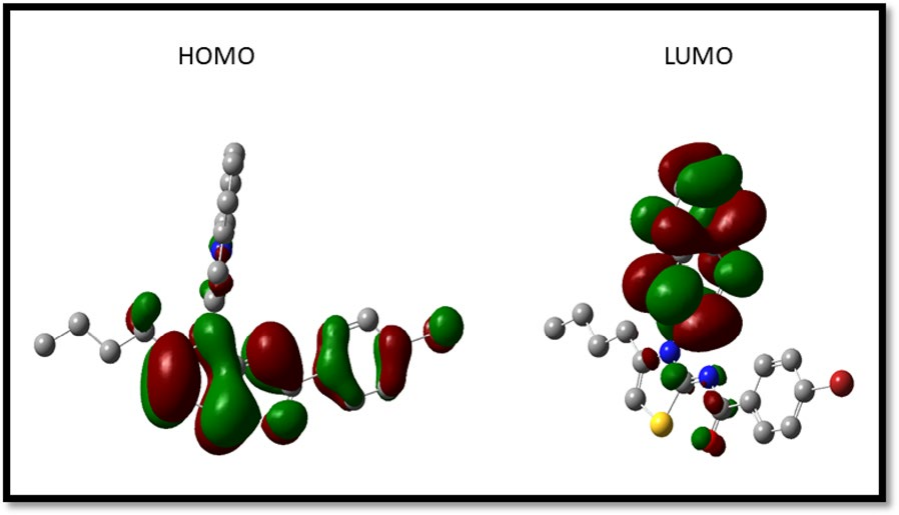
FMOs analysis of compound 6

The FMOs analysis was established to determine the localization of HOMO and LUMO orbitals on compound 6. The maximum HOMO delocalization was observed at the five membered hetrocyclic ring while two fused benzene ring show no delocalization, a reciprocal trend was observed in case of LUMO orbitals. FMOs are illustrated in Fig. 9.

The global and local reactivity descriptors are prime analytical metrics in regulating the chemical reactivity profile of the compound. Various descriptors including ionization energy, electron affinity, electronegativity and electrophilicity contributed substantially toward reactivity of the compound. As tabulated below, it can be observed that compound possessed ionization energy of 0.2165 with comparable electron donating power of 0.234. Whereas, electron accepting and elecrophilicity index is lower than electron donating power which means compound donate electrons readily. The reactivity descriptors of compound 6 is mentioned in Table 4.

**Table 4:** Reactivity descriptors of compound 6

Compound	Ionization energy	Electron affinity	Electron donating power	Electron accepting power	Net electrophilicity	Electro-negativity	Electrophilicity index
6	0.2165	0.07583	0.234	0.088	0.321	0.146	0.152

### Elastase inhibition assay

**Fig. 10:**
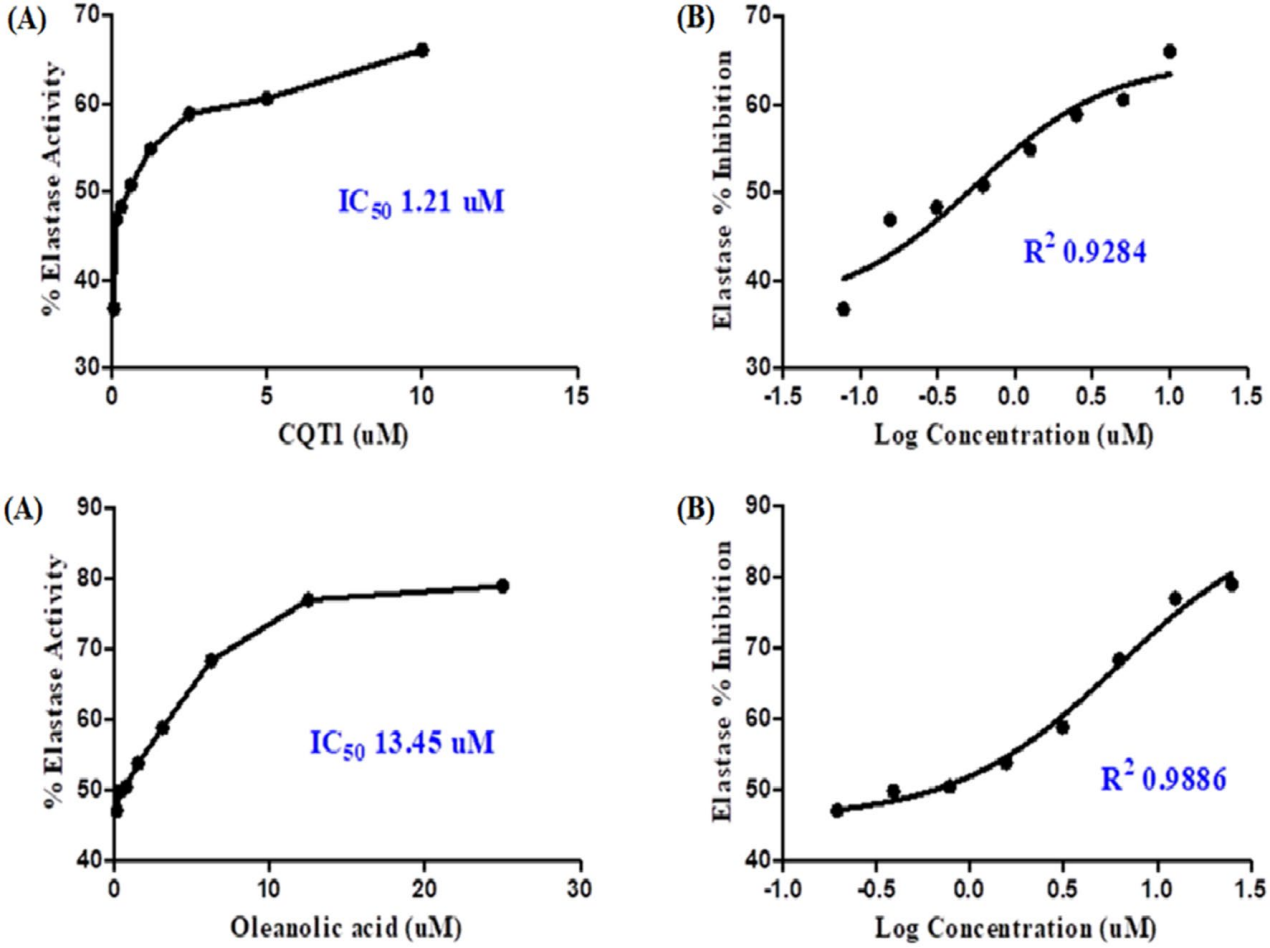
IC_50_ Curves of quinolinyl iminothiazoline 6 and Oleanolic Acid. **A** The concentrations of quinolinyl iminothiazoline 6 and Oleanolic Acid. **B** Curves of log concentrations vs Inhibition % of quinolinyl iminothiazoline 6 and Oleanolic Acid. IC_50_ value was calculated by Graph Pad Prism 5.0 using nonlinear regression [41]

Elastase from porcine pancreas was used to investigate the inhibitory potential of the titled compound. The titled compound showed good inhibitory potential against elastase i.e. IC_50_ 1.21 µM as compared to the oleanolic acid with an IC_50_ value of 13.45 µM which has also been reported earlier [37–40] and is shown in Fig. 10.

### Molecular docking studies

Molecular docking studies have been widely used to predict the binding affinity of ligands against target proteins. In this study, we performed molecular docking studies to investigate the binding affinity of the synthesized crystal and the standard quercetin against the elastase protein. Elastase enzyme is an important serine protease that plays a crucial role in a variety of physiological processes, including tissue remodeling, inflammation, and host defense. However, its dysregulation has been associated with a number of diseases, such as emphysema, chronic obstructive pulmonary disease (COPD), and cystic fibrosis. Therefore, the development of inhibitors that can selectively target and regulate elastase activity has become an area of great interest in the pharmaceutical industry.

As a result of the findings, it was demonstrated that compound 6 and the quercetin could dock into the active pocket of the elastase protein. Compared to the standard quercetin, which showed a docking value of − 7.2 kcal/mol, the crystal displayed a higher docking score of − 7.4 kcal/mol. This suggests that the crystal has a greater ability to suppress the elastase enzyme's activity. The docking outcomes also showed that the crystal exhibited a number of non-covalent contacts, such as hydrogen bonds and hydrophobic interactions, with the active site residues of the elastase protein. These interactions might help explain why the crystal has a higher affinity for the elastase protein. In conclusion, the molecular docking experiments gave important information on the binding affinity and possible inhibitory activity of the produced crystal against the elastase protein, and they may help create new inhibitors for the treatment of disorders associated with elastase. The molecular interactions between the synthetic crystal and the standard against the elastase enzyme are shown in Table 5 below.

**Table 5:** The molecular interactions of 6 and standard quercetin

Compound	Docking Score (kcal/mol)	Hydrogen bonding residues	Bond length of hydrogen bonding (Angstroms)	Hydrophobic residues
Crystal (6)	− 7.4	Gln34	3.1	Asn76, Glu80, Thr75, Asn74, Ser37, Tyr38, Leu73, His40
Quercetin	− 7.2	Asn188, Asn132, Ser162, Trp159	3.14, 2.71, 2.84, 3.22	Gly187, Gly184, Pro161, Trp171, Val163, Asn133

**Fig. 11:**
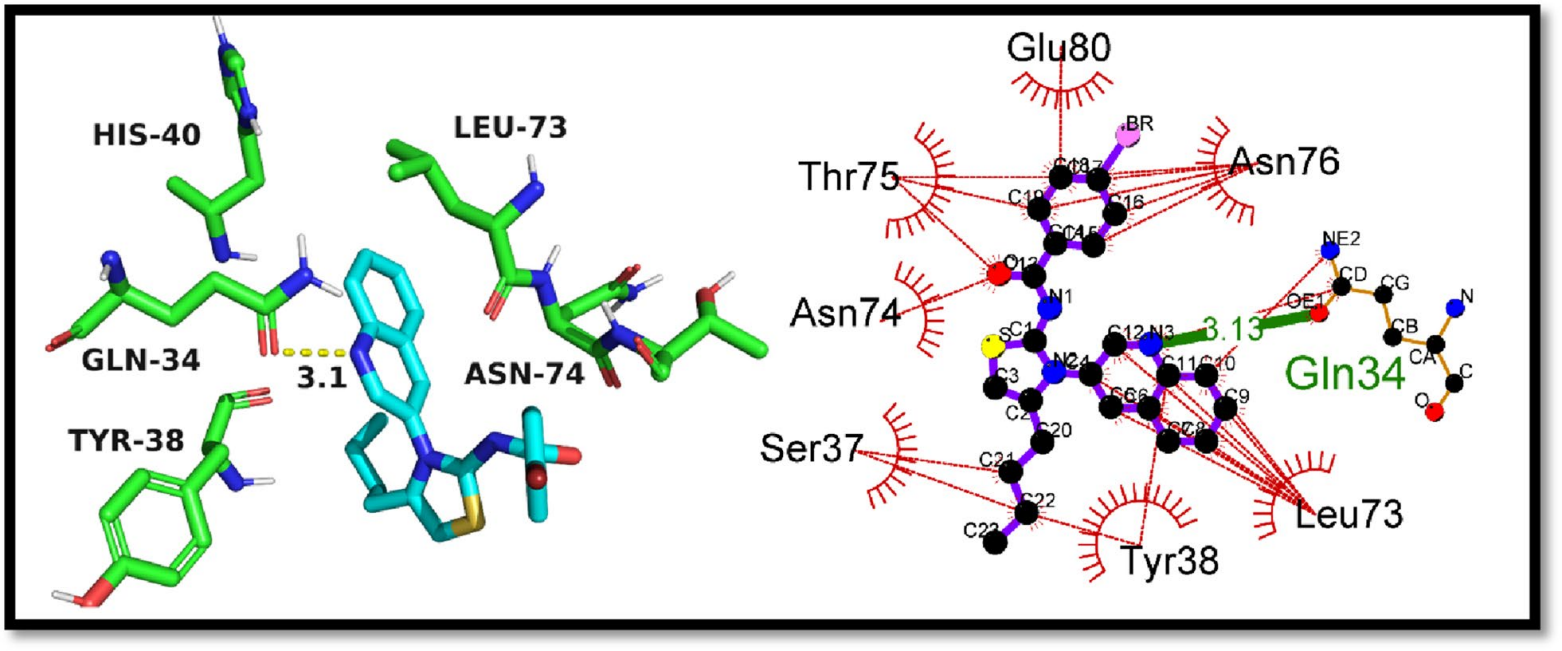
The putative 2D and 3D binding mode of crystal (6) against elastase enzyme

The crystal that was created (6) was docked against the elastase enzyme utilizing molecular docking studies, and the results showed that the crystal had a docking score of − 7.4 kcal/mol. In the process of analyzing the results of the docking, it was found that the crystal established hydrogen bonds with the Gln34 residue of the elastase enzyme. The bond length of these bonds was 3.1 Å. In addition to this, the crystal was found to have interactions with hydrophobic residues such as Asn76, Glu80, Thr75, Asn74, Ser37, Tyr38, and Leu73. The formation of hydrogen bonds and the presence of hydrophobic contacts in the crystal-elastase complex are both indicators that the molecule that was synthesized has the potential to serve as a powerful inhibitor of the elastase enzyme. Figure 11 depicts the probable 2D and 3D modes of crystal inside the active pocket of elastase.

**Fig. 12:**
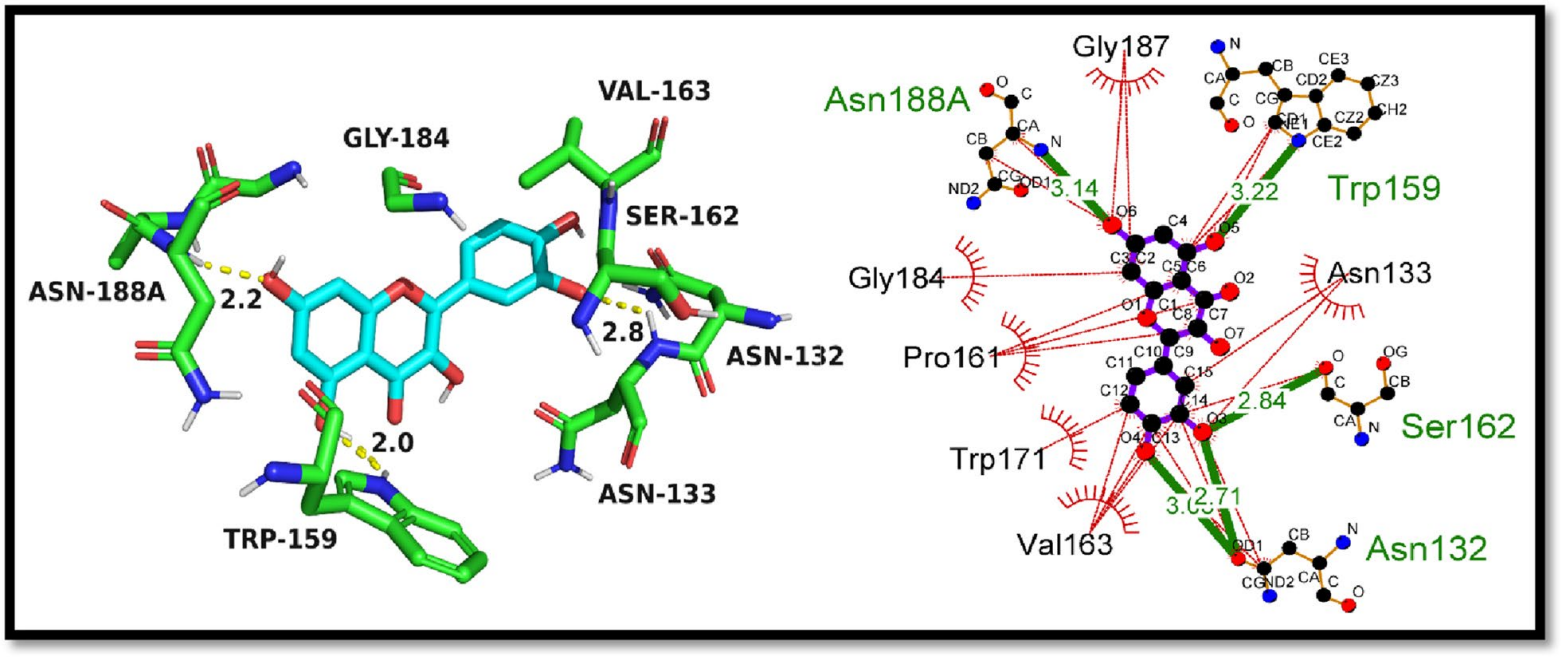
The putative 2D and 3D binding mode of quercetin against elastase enzyme

The standard compound, quercetin, was also subjected to molecular docking against the elastase enzyme, and it exhibited a docking score of − 7.2 kcal/mol. The hydrogen bonding residues for quercetin were Asn188, Asn132, Ser162, and Trp159, with respective bond lengths of 3.14, 2.71, 2.84, and 3.22 Å. The hydrophobic residues involved in the binding were Gly187, Gly184, Pro161, Trp171, Val163, and Asn133. These findings suggest that quercetin produce stable interactions but synthesized crystal (6) had outperformed the standard in terms of binding affinity. The findings of in-silico investigations were also supported by in-vitro studies. So it can be deduced that crystal (6) may also be a potential inhibitor of elastase, as it displayed strong binding affinity and hydrogen bonding interactions with key residues in the active pocket of the enzyme. Figure 12 is illustrating the putative 2D and 3D mode of quercetin inside active pocket of elastase.

### Molecular dynamics simulation

Molecular dynamics (MD) simulations are an important tool for investigating the thermodynamic properties of biological systems in physiological conditions. The stability of complex (protein–ligand) was evaluated by using the Desmond software. The best docked conformation was used as the starting structure, and the simulation was run for 100 ns. The deep understanding of the complex behavior was evaluated by root mean square deviation; RMSD, rot mean square fluctuation; RMSF, contact and ligand interaction profile. The data obtained from these matrices enabled us to examine the complex's structural stability and dynamic behavior over time, providing valuable insights into the protein–ligand interaction.

**Fig. 13:**
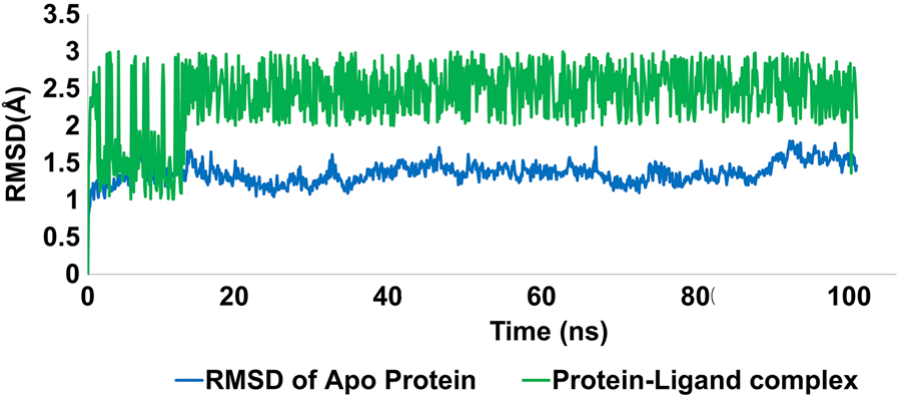
Evolution of RMSD for protein (C alpha blue colored trajectory), protein–ligand complex (green colored trajectory)

Figure 13 displays the RMSD graphical presentation of the protein C-alpha atoms (backbone) and protein–ligand complex. The C-alpha atoms were stable, with small window fluctuations ranging from 1 to 1.7 Å. However, fluctuations of up to 1.8 Å were observed in some amino acid residues, such as Gly12, Pro17, and Tyr122. The protein C alpha atoms had an average RMSD within the acceptable limit of 1.36 Å which is quite acceptable. These observations indicate the significant stability of the protein and the fewest conformational changes during MD simulations. On the other hand, the protein–ligand complex exhibited slight perturbations for the initial 10 ns, during which the complex fluctuated around 1.2–2.8 Å; these fluctuations got stable after 20 ns of MD simulations. It was notable that significant contacts were observed with active-site residues. Despite a few rearrangements, the ligand remained attached to the active site and didn’t flip outside of the active pocket. The equilibration of the trajectory after 20 ns might be due to establishing significant contacts with Tyr39, Tyr38, Arg65, Asn74, and Leu151. These contacts stabilized the protein–ligand complex and retained an average RMSD of 2.40 Å, which is acceptable. In summary, Fig. 13 provides valuable information on the stability of the complex under the simulation conditions, indicating the ligand's ability to bind to the active site of the enzyme.

**Fig. 14:**
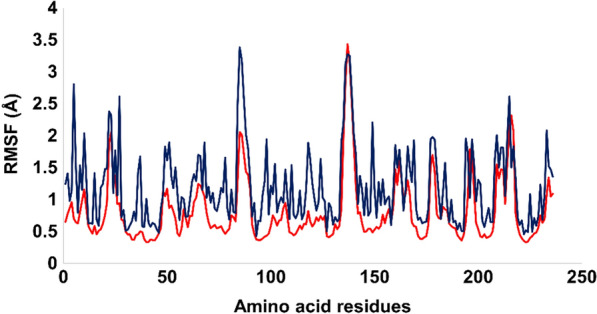
The RMSF peaks for Apo protein (dark blue peaks) and liganded protein (red colored peaks)

This study employed RMSF analysis to generate the fluctuation profiles for all residues, revealing optimal fluctuations for the majority of amino acid residues. However, it was observed that residues Thr147, Gly148, Gly240, Ile241, and Met242 exhibited slightly higher fluctuations for more than 1 angstrom, whereas the remaining residues were significantly more stable during MD simulations. These amino acid residues belong to terminal regions, which are less rigid and tend to be more flexible. In addition, the amino acids in contact with the ligand were significantly stable, particularly the amino acid residues ranging from 20 to 50 that were in contact with the ligand and exhibited RMSF values of less than 1 angstrom, which demonstrate the significant stability pattern. The average RMSF value for the entire protein was found to be 1.19 Å, which is well within an acceptable limit. In addition, the RMSF data for liganded protein was also retrieved which revealed that ligand bound protein got more stable with average RMSF value of 0.81 Å. In addition, fluctuations of each residues was also got stable after binding of lignad with targeted protein. Figure 14 presents the RMSF evolution plot, with rectangle box indicating the specific amino acid residues that were in contact with the ligand. This analysis provides valuable insights into the local flexibility and dynamics of the protein, which can be useful for understanding its function and interactions with other molecules.

**Fig. 15:**
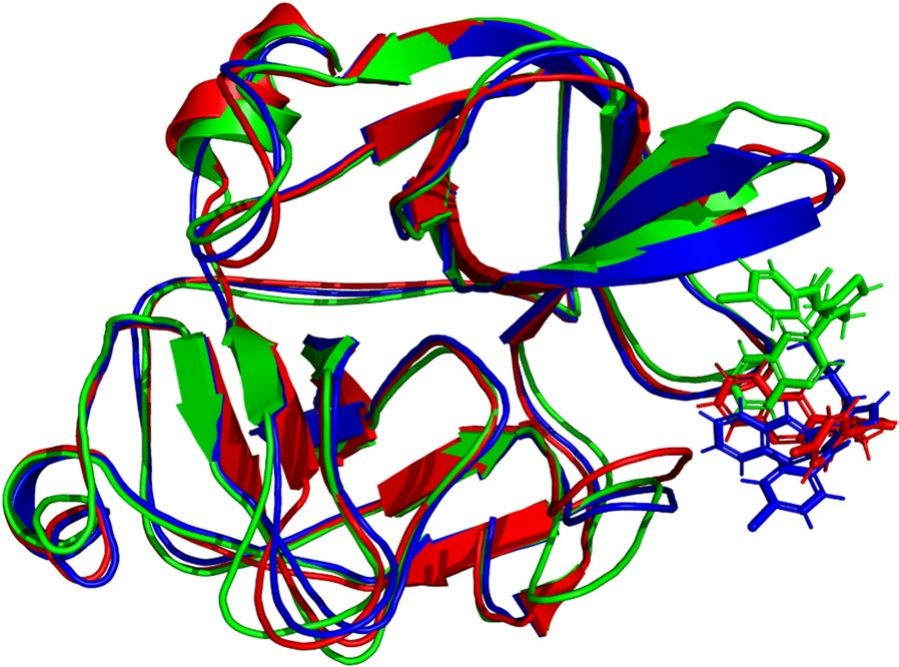
Superposition of Protein–ligand complex at various intervals. The green colored indicating the complex at 0 ns, Blue color indicate position of complex at 50 ns and red color is indicating the position of complex at 100 ns

The stability of protein ligand complex and binding of ligand with active site residues was further validated by taking the snapshot of MD simulation trajectory at different intervals and superposition these snapshots with respect to reference frame taken at 0 ns. It was notable that ligand remained attached to active site and didn’t exhibited flipping at any interval i.e., during initiation, middle and end of the trajectory. However slight rearrangements of ligand did observed during initiation and terminal stages of simulated trajectory. Figure 15 is illustrating the snapshots of simulated trajectory at different intervals.

**Fig. 16:**
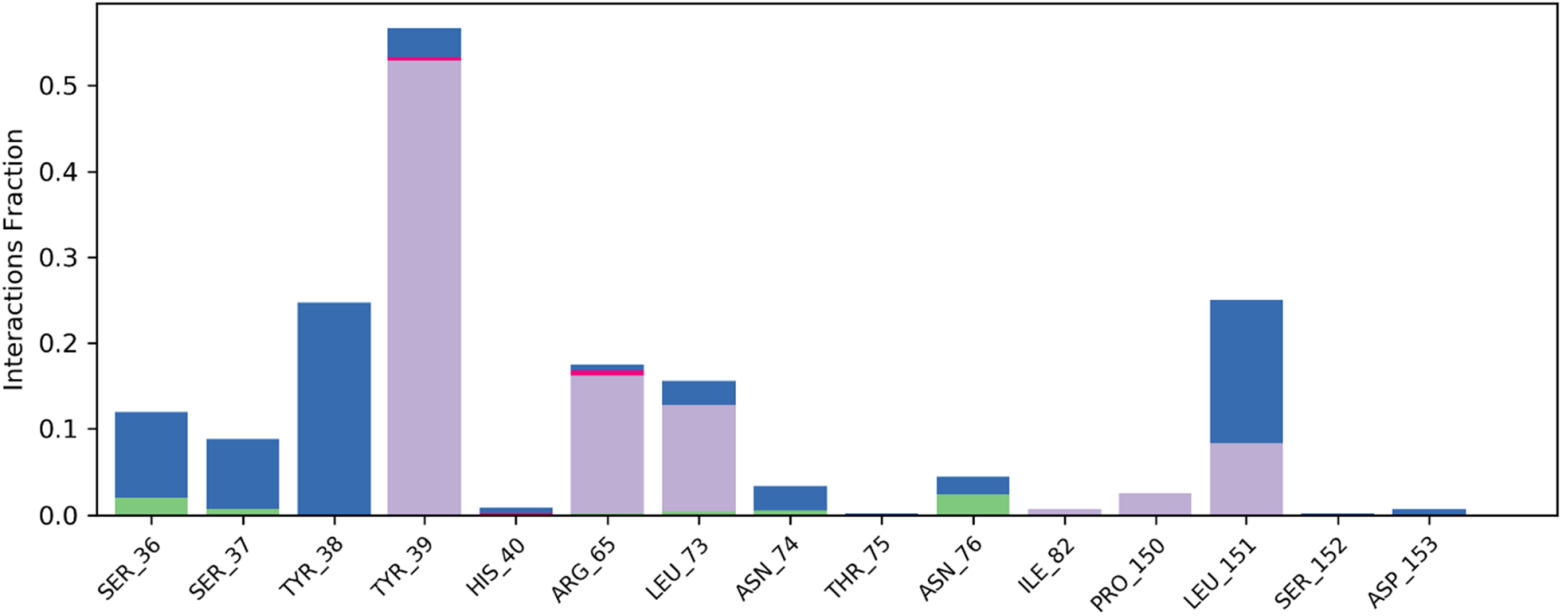
Contact map of synthesized crystal with targeted protein observed during MD simulation

Ligand contact profile is an important analytical metric to determine the efficiency of protein–ligand interactions. It was notable that strong interactions were observed with active site residents. Especially, Tyr39, Arg65, Leu73, Leu151, and Pro150 were engaged in hydrophobic interactions for more than 10% of the simulation time, and the interaction fraction was 50% for Tyr39. In addition, water bridges and hydrogen bonding were also stabilizing the protein ligand complex. Figure 16 illustrates the detailed contact map profile of the ligand with protein.

**Fig. 17:**
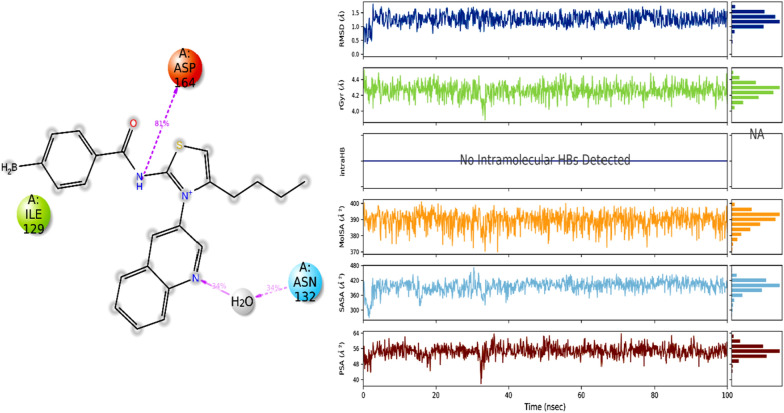
(Left) Interaction profile of ligand with specific amino acid residues, (right) ligand properties

Figure 17 presents the interaction profile and ligand properties of the protein–ligand complex. The analysis reveals that the amino acid residue ASP164 was strongly occupied by ligand atoms, with a contact time of over 70% during the simulation. The second most occupied residue was ILE129, indicating a significant contribution to the stability of the complex.

**Fig. 18:**
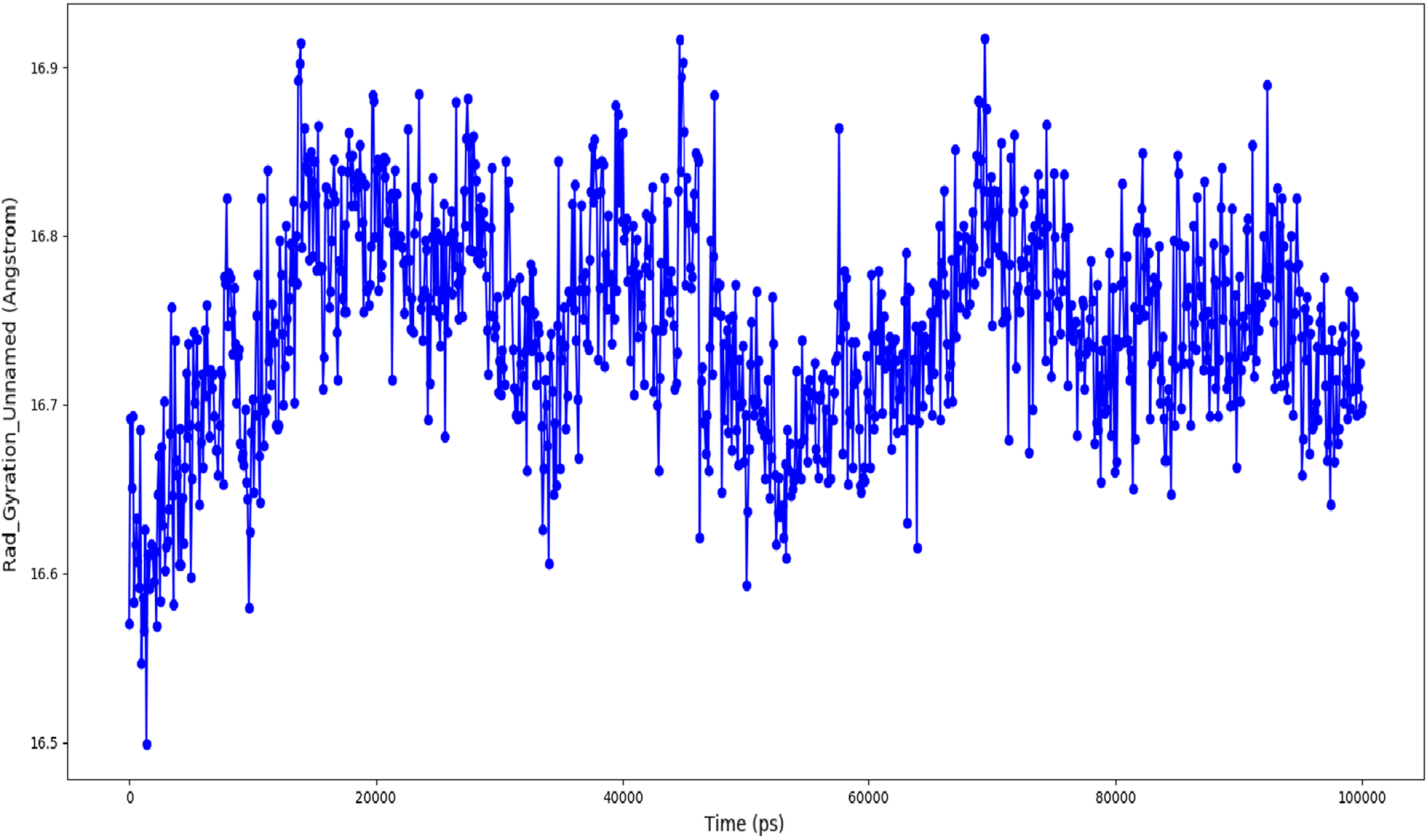
Radius of gyration

The radius of gyration (Rg) is a critical analytical metric that helps determine the center of mass and compactness of proteins. A low Rg value indicates that the protein is highly compact with fewer structural changes, while a high Rg value suggests poor stability and more structural variations in the protein. Here, the protein showed very few structural changes with Rg ranges between 16.6 and 16.9 Å, indicating that the protein's mass was evenly distributed around a single point and remained compact throughout the simulated trajectory. Figure 18 depicted the Rg of the studied protein.

**Fig. 19:**
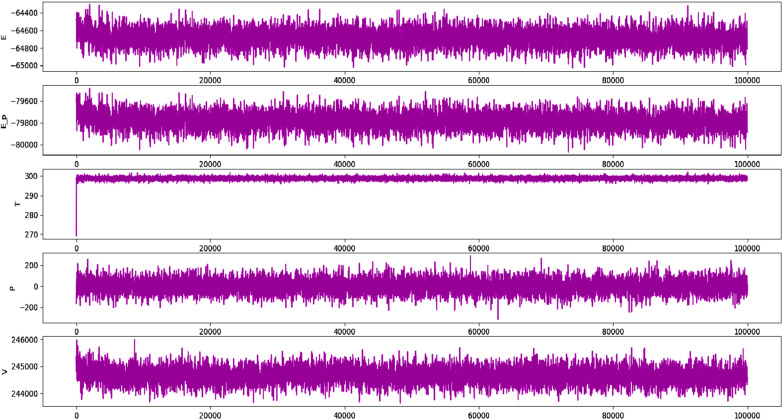
Electrostatic potential, optimization energy and equilibrated temperature, volume and pressures

The MD simulation studies were conducted under constant pressure and temperature conditions using different barostat and thermostat for maintenance of pressure and temperature. It was observed that system was equilibrated constant pressure of 1.01 bar pressure and at constant temperature of 300 K. The electrostatic energy and equilibrated temperature and pressure is illustrated in Fig. 19.

## Materials and methods

### Experimental

#### Synthesis of (*Z*)-4-bromo-*N*-(4-butyl-3-(quinolin-3-yl)thiazol-2(3*H*)-ylidene)benzamide (6)

In order to make the acyl thiourea product, a solution of potassium thiocyanate (1 mmol) was combined with dry acetone (15 mL) in a round-bottom flask that had a reflux condenser attached to it. This step was carried out in a reflux apparatus. After this, the mixture was swirled for a total of five minutes. After that, a 1 mmol solution of pentanoyl chloride that had been dissolved in acetone was added to the reaction mixture in a dropwise manner. In order to produce the isothiocyanate, the mixture was subjected to heating at a temperature that produced reflux for three to four hours. Following the step of cooling, an acetone solution containing one millimole of 3-aminoquinoline was added slowly, and the temperature was kept at 60 °C for 12–14 h in order to generate the acyl thiourea product. After this step, the reaction mixture was cooled using water that had been chilled with ice, and the result was separated using filtration. In order to obtain the finished product, the crude product was recrystallized in ethanol in order to undergo purification.

**Fig. 20:**
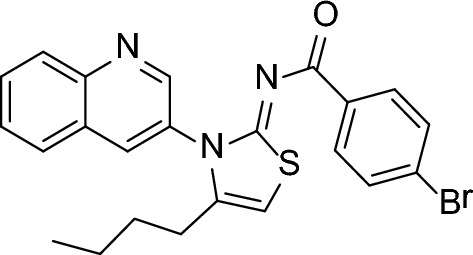
(*Z*)-4-bromo-*N*-(4-butyl-3-(quinolin-3-yl)thiazol-2(3*H*)-ylidene)benzamide (6)

To synthesize quinolinyl-iminothiazoline 6, acyl thiourea (1 mmol) was mixed with dry dichloromethane (15 mL) and triethyl amine (1 mmol) in a round bottom flask. After heating the combination to 50 °C for 24 h in a nitrogen environment, a *p*-bromophenacyl bromide solution containing one millimole of *p*-bromophenacyl bromide was added to it over the course of 30 min. Following the use of TLC to validate that the reaction was successful, the mixture was filtered, and a rotary evaporator was utilized to remove the solvent. As can be seen in Fig. 20, the product that was obtained underwent additional purification by being recrystallized in ethanol.

Brown crystalline; M.P = 173 °C; yield = 75%; R_f_ = 0.45 (EtOAc: n-hexane, 2:8); FT-IR (ATR) in cm^−1^, 3059 (H-C, Ar), 2957 (H-C, thiazoline), 2928, 2858 (H-C, alkyl asymmetric and symmetric), 1738 (C=O), 1589 (C=C, Ar); ^1^H-NMR (300 MHz, CDCl_3_); in δ (ppm), 8.93 (d, 1H, *J* = 2.1 Hz, quinolinyl-H), 8.32 (d, 1H, *J* = 8.4 Hz, quinolinyl-H), 8.23 (d, 1H, *J* = 2.4 Hz, quinolinyl-H), 7.91 (m, 2H, quinolinyl-H), 7.82 (d, 2H, *J* = 8.4 Hz, phenyl-H), 7.70 (m, 1H, quinolinyl-H), 7.41 (d, 2H, *J* = 8.4 Hz, phenyl-H), 6.45 (s, 1H, thiazoline-H), 2.36 (t, 2H, *J* = 7.2 Hz, aliphatic-H), 1.53 (m, 2H, aliphatic-H), 1.2 (m, 2H, aliphatic-H), 0.90 (t, 3H, *J* = 7.5 Hz, aliphatic-H); ^13^C-NMR (75 MHz, CDCl_3_) in δ (ppm), 172.5 (C=O), 171.0 (imine, C), 149.2, 148.1, 137.3, 134.6, 133.8,130.9, 129.8, 129.4, 128.5, 127.5, 127.0, 126.9, 126.2, 105.0 (thiazoline, C), 31.2, 22.5, 19.2, 13.2. Anal. Calc. for C_23_H_20_BrN_3_OS: C, 59.23; H, 4.32; N, 9.01; Found: C, 59.21; H, 4.31; N, 9.00.

### X-ray crystallography

The crystallographic data for compound (I) was collected using Mo Kα radiation (λ = 0.71073 Å) on the Rigaku Oxford Diffraction diffractometer. The data was processed using the SHELX program packages SHELXL [42] and SHELXL [43] for structure solving and refining [44], while ORTEP-3 [45] and PLATON [46] were used for drawings. To correct for multi-scan absorption, CrysAlis PRO 1.171.38.46 [6] was applied. The positions of the hydrogen atoms were calculated using a riding model and were refined by applying the constraints of Uiso(H) = k X Ueq (C, O), where k = 1.5 for CH3 hydrogens and k = 1.2 for other H atoms. The distances for the hydrogen atoms were set at 0.95 Å (for aromatic CH), 0.99 Å (for CH2), and 0.98 Å (for CH3). The reported crystallographic data for the structure has been deposited with the Cambridge Crystallographic Data Centre (CCDC No. 2221961), which can be obtained by contacting CCDC at 12 Union Road, Cambridge CB2 1EZ, UK (fax: + 44 1223 336033 or e-mail: deposit@ccdc.cam.ac.uk or at http://www.ccdc.cam.ac.uk).

The crystal data for the titled compound C_23_H_20_BrN_3_OS was obtained through a series of experiments. The chemical formula of the compound was determined to be C_23_H_20_BrN_3_OS with a molecular weight of 466.39. The crystal system was found to be triclinic with the space group P − 1, and the temperature used for the experiment was 173 K. The crystal dimensions were measured and found to be 9.2304 (6) Å, 11.1780 (8) Å and 11.3006 (6) Å for a, b, and c, respectively. The angles α, β, and γ were 107.146 (5)°, 93.701 (5)° and 110.435 (6)°, respectively, with a volume of 1025.61 (12) Å^3^ and a Z value of 2. The diffraction data were collected using the Rigaku Oxford Diffraction diffractometer and the empirical absorption correction was carried out using the CrysAlis PRO 1.171.38.46 software. The scaling algorithm used for absorption correction was SCALE3 ABSPACK. The Tmin and Tmax were 0.842 and 1.000, respectively, and a total of 12,759 reflections were measured, out of which 6744 were independent and observed with I > 2σ(I). The R_int_ value was found to be 0.030 and (sin θ/λ)_max_ was 0.761 Å^−1^. In terms of refinement, the H-atom parameters were constrained and the Δρ_max_ and Δρ_min_ were 0.59 and − 0.54 e Å^−3^, respectively. The R[F^2^ > 2σ(F^2^)], wR(F^2^), and S values were 0.042, 0.090, and 1.02, respectively, with a total of 263 parameters used. Overall, the crystal data provides a detailed insight into the structural properties of the titled compound and lays the foundation for further exploration and study of its properties.

### Hirshfeld surface studies

The Crystal Explorer 17.5 software [47, 48] was used for the conduction of HS analysis and for the determination of intermolecular interactions. From the nearest nucleus, the HS distances d_i_ and d_e_ were determined, and D_norm_ was used to estimate the normal contact distance, which was illustrated by the colors red, white, and blue. The white surface displayed contacts at distances that were equivalent to the sum of the van der Waals radii [49], but the red and blue colors indicated distances that were shorter or longer than the van der Waals radii, respectively. Bright-red patches indicated either a donor or an acceptor role, while blue and red regions correlated to positive and negative potentials on the HS when mapped over electrostatic potential [50]. The stacking of π … π was made clear by the HS shape-index, which displayed red and blue triangles that were near to one another. An overall two-dimensional fingerprint plot and its many delineations were used to depict the relative contributions of various interactions to the Hirshfeld surface [51, 52]. This plot was used to highlight the relative contributions of different interactions. The different types of interactions are given in the Fig. 6, followed by H/H interactions. The distribution of points on the Hirshfeld surface reflected the relative contributions of each interaction [31]. In accordance with the findings of the investigation of the Hirshfeld surface, the crystal packing is established by the interactions between hydrogen atoms. The predominance of H…H and H…C/C…H interactions gives rise to the hypothesis that hydrogen bonding and van der Waals interactions are the preponderant driving factors in the crystal packing [53].

### Density functional theory (DFTs) studies

The optimization of the molecular structure of the synthesized compound was conducted using Density Functional Theory (DFT) calculations [54]. Specifically, the B3LYP method was employed, which is a popular DFT functional used for predicting molecular geometries, vibrational frequencies, and other molecular properties. The cc-pVDZ basis set [55] was selected for basis set construction, which is a high-quality basis set that includes polarization and diffuse functions. This basis set is known to accurately describe molecular properties such as bond lengths, angles, and dipole moments. The optimization process involved finding the minimum energy conformation of the molecule by varying the atomic positions until a stable geometry was reached [56]. The electronic structure of the compound was examined with the Gaussian 09 software in order to further investigate the chemical characteristics of the compound. In order to explore the chemical reactivity of the molecule, the frontier molecular orbitals were studied [54]. These orbitals represent the highest occupied molecular orbital (HOMO) and the lowest unoccupied molecular orbital (LUMO), respectively. In addition, the chemical hardness and softness were computed based on the energy gap between the HOMO and the LUMO. This provided insight into the stability of the complex as well as its capacity to react with other molecules. These calculations were carried out by utilizing well-known quantum chemistry methods, which provided a greater comprehension of the characteristics of the compound as well as its possible uses [57]. Gauss View 6 was used to do visualization and analysis on each and every optimized output file [58].

### Elastase inhibition assay

We used a modified approach to assess elastase inhibition [59, 60]. Elastase from the pig pancreas was utilized to measure p-nitroaniline liberated from the substrate (*N*-succinyl-Ala-Ala-Ala-*p*-nitroanilide) to assess inhibition. The detailed protocol is given in our previously published article [61] and also given in the Additional file 1.

### Molecular docking methodology

**Fig. 21:**
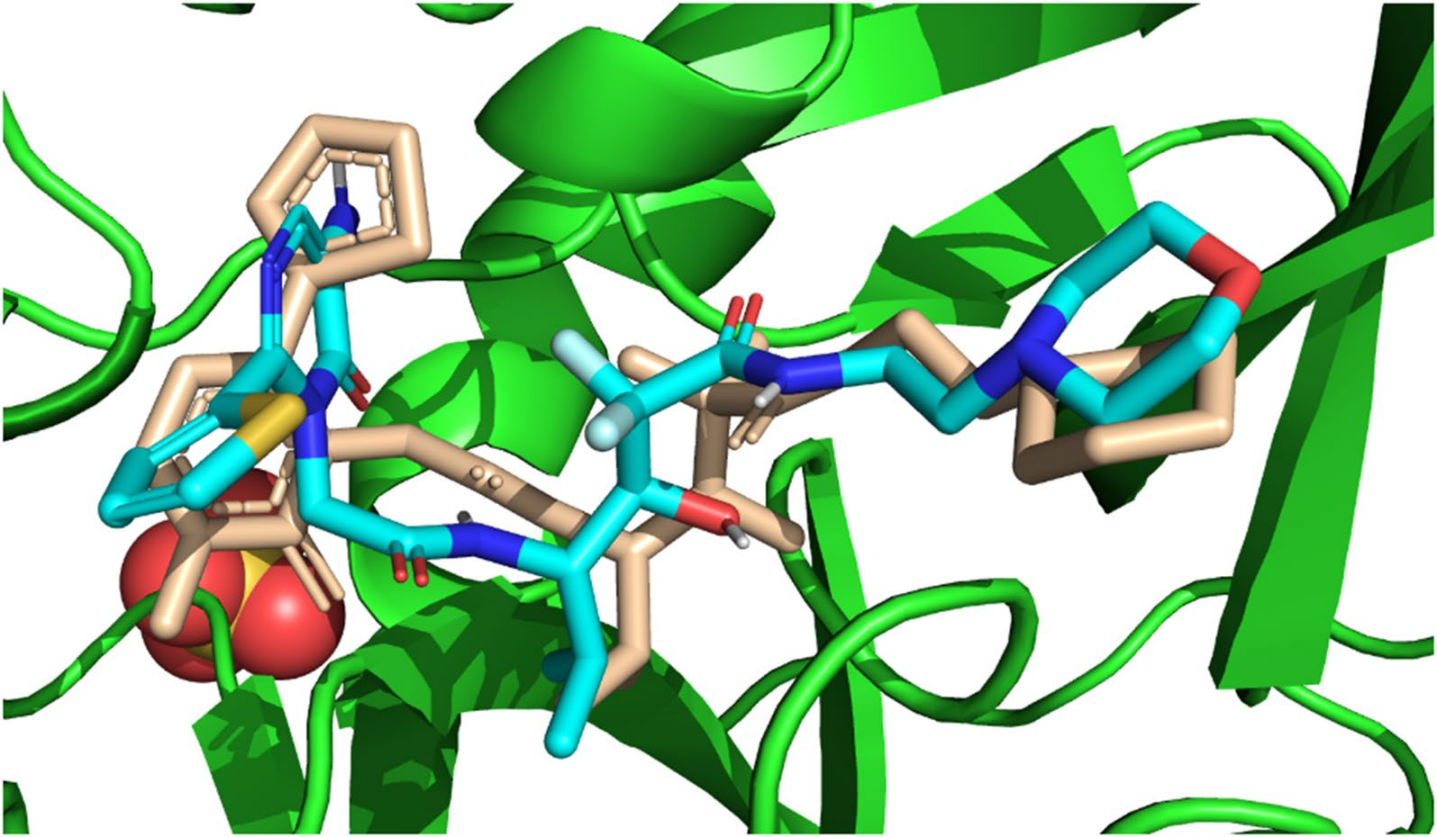
Illustration of docking protocol validation. Blue ligand is regenerated pose whereas pink ligand is native conformation (RMSD 2.3 angstroms)

Molecular docking is an efficient approach to study these interactions between ligands and proteins [62]. In our study, we used molecular docking to evaluate the potential molecular interactions of a synthesized compound with elastase enzyme. Initially, we retrieved the elastase protein with the PDB ID: 1EAU (porcine pancreatic elastase) from the protein data bank [63] (https://www.rcsb.org/) [64]. The retrieved protein was prepared using the MGL tools preparation wizard, which involved the removal of hetero atoms, addition of polar hydrogens, and gasteiger charges. Missing residues were also prepared using MGL tools. Subsequently, the synthesized compound was subjected to preliminary energy minimization using ChemDraw 3D and saved in the desired format for docking using AutoDock Vina [65, 66]. To carry out the docking process, a grid box with dimensions of 12.758 Å, 47.649 Å, and 84.536 Å in the x, y, and z dimensions, respectively, was generated at the active pocket of the elastase enzyme with a spacing of 0.5 Å. The Genetic algorithm was used to retrieve 100 poses for the synthesized crystal. The conformation with the best binding affinity and RMSD value was selected and analyzed using PyMOL [67] and LigPlot plus [68]. Additionally, the stability of the selected conformation was evaluated under accelerated conditions using molecular dynamics simulations. The co-crystal ligand of the elastase enzyme was re-docked into the active pocket (Fig. 21) in order to evaluate the docking process. The RMSD value was found to be about 2, supporting the reliability of the docking protocol [69].

### Molecular dynamics simulation studies

To analyze the interaction between protein and ligand, Desmond software was used where a 100 ns simulation of protein–ligand complex was carried out using the TIP3P solvent model. The optimized potential for liquid simulations (OPLS3) forcefield [70] was employed under periodic boundary conditions [71]. The system was solvated with TIP3P water [72] molecules in an orthorhombic solvation box. To neutralize the system, NaCl ions were added, resulting in a concentration of 0.15 M. All the conditions were set as discussed earlier [66, 73] were employed. The simulation was performed for 100 ns in triplicate with snapshots of the trajectories saved every 100 ps and a time step of 2 fs. For accurate investigation of electrostatic interactions, the particle mesh Ewald method [74] was utilized. The Desmond simulation interaction diagram [75] protocol was employed to analyze the protein–ligand complex trajectories as reported earlier [66].

## Conclusion

In conclusion, the current study demonstrated the successful synthesis and characterization of a structurally varied molecule known as quinolinyl iminothiazoline. This compound demonstrated significant activity against the elastase enzyme. The synthesized derivative had substantial activity in an in vitro elastase inhibition assay, with an IC50 value of 1.21 µM. This value indicated that the synthesized derivative was more potent than the standard, which was oleanolic acid, which had an IC50 value of 13.45 µM. The kinetic analyses provided additional confirmation that the chemical possesses remarkable binding energies as well as a competitive method of inhibition. The in silico studies including x-ray analysis, HS analysis, crystal void analysis, DFTs, molecular docking, and MD simulations supported the experimental findings by demonstrating the potential molecular interactions and stability of the protein–ligand complex. Furthermore, the x-ray analysis provided insights into the structural properties of the compound, revealing planar rings positioned at dihedral angles, and the molecules in the compound forming infinite double chains through hydrogen bonds and π-π interactions, creating a three-dimensional architecture. These findings altogether indicate that the synthesized compound holds strong potential as a lead compound for the development of a new drug against elastase enzyme.

### Supplementary Information

**Additional file 1**. **Table S1** Experimental details. Spectroscopic characterization data. Elastase inhibition assay.

## Data Availability

Data will be available on request by the corresponding author.
