# Supplementary material for: Synthesis, DFT and molecular docking of novel (Z)-4-bromo-N-(4-butyl-3 (quinolin-3-yl)thiazol-2(3H)-ylidene)benzamide as elastase inhibitor
Source: BMC Chem. 2023 Aug 7;17(1):95. doi: 10.1186/s13065-023-00985-4 (PMC10408170; doi:10.1186/s13065-023-00985-4)
Supplement: Supplementary file 1 — Additional file 1. Table S1 Experimental details. Spectroscopic characterization data. Elastase inhibition assay. [file 13065_2023_985_MOESM1_ESM.docx]

**Synthesis, DFT and Molecular Docking of Novel (*Z*)-4-bromo-*N*-(4-butyl-3 (quinolin-3-yl)thiazol-2(3*H*)-ylidene)benzamide as Elastase Inhibitor**

Muhammad Naeem Mustafa^a^, Pervaiz Ali Channar^b^, Syeda Abida Ejaz^c*^, Saira Afzal^d^, Mubashir Aziz^c^, Tahira Shamim^e^, Aamer Saeed^a*^, Aisha A.Alsfouk^f^, Rabail Ujan^g^, Qamar Abbas^h,i^, Tuncer Hökelek^j^

^a^ Department of Chemistry Quaid-i-Azam University-45320, Islamabad, Pakistan

*^b^ Department of Basic sciences and Humanities, Faculty of Information Sciences and Humanities, Dawood University of Engineering and Technology Karachi 74800, Pakistan*

*^c^ Department of pharmaceutical chemistry, Faculty of pharmacy, The Islamia university of Bahawalpur, Bahawalpur,63100, Pakistan.*

*Faculty of Pharmacy, The University of Lahore, Lahore, Pakistan*

*^e^ University College of Conventional Medicine, Faculty of Medicine & Allied Health Sciences, The Islamia University of Bahawalpur, Bahawalpur 63100, Pakistan*

*^f^Department of Pharmaceutical Sciences, College of Pharmacy, Princess Nourah bint Abdulrahman University P.O Box 84428, Riyadh 11671, Saudi Arabia*

*^g^Dr. M. A. Kazi Institute of Chemistry, University of Sindh, Jamshoro, Pakistan*

^h^ Department of Biology, College of Science, University of Bahrain, Sakhir Campus, Sakhir, 32038, Bahrain

*^i^ College of Natural Sciences, Department of Biological Sciences, Kongju National University, Gongju-32588, Republic of Korea.*

*^j^Department of Physics, Faculty of Engineering, Hacettepe University, Beytepe-Ankara, 06800, Turkey*

**Corresponding author:** Aamer Saeed, Department of Chemistry Quaid-i-Azam University-45320, Islamabad, Pakistan*.*  [aamersaeed@yahoo.com](mailto:aamersaeed@yahoo.com). Syeda Abida Ejaz, Department of Pharmaceutical Chemistry, Faculty of Pharmacy, The Islamia University of Bahawalpur, Bahawalpur 63100, Pakistan. [abida.ejaz@iub.edu.pk](mailto:abida.ejaz@iub.edu.pk)

**Table 1. Experimental details**

| Crystal data | |
| --- | --- |
| Chemical formula | C_23_H_20_BrN_3_OS |
| *M*_r_ | 466.39 |
| Crystal system, space group | Triclinic, *P -*1 |
| Temperature (K) | 173 |
| *a*, *b*, *c* (Å) | 9.2304 (6), 11.1780 (8), 11.3006 (6) |
| α, β, γ (°) | 107.146 (5), 93.701 (5), 110.435 (6) |
| *V* (Å^3^) | 1025.61 (12) |
| *Z* | 2 |
| Radiation type | Mo *K*α |
| µ (mm^−1^) | 2.13 |
| Crystal size (mm) | 0.24 × 0.22 × 0.18 |
|  | |
| Collection of data | |
| Diffractometer | Rigaku Oxford Diffraction |
| Absorption correction | Multi-scan *CrysAlis PRO* 1.171.38.46 (Rigaku Oxford Diffraction, 2015) Empirical absorption correction using spherical harmonics, implemented in SCALE3 ABSPACK scaling algorithm. |
| *T*_min_, *T*_max_ | 0.842, 1.000 |
| Number of measured, independent and observed [*I* > 2σ(*I*)] reflections | 12759, 6744, 5058 |
| *R*_int_ | 0.030 |
| (sin θ/λ)_max_ (Å^−1^) | 0.761 |
|  | |
| Refinement | |
| *R*[*F*^2^ > 2σ(*F*^2^)], *wR*(*F*^2^), *S* | 0.042, 0.090, 1.02 |
| Total reflections | 6744 |
| Total parameters | 263 |
| H-atom treatment | H-atom parameters constrained |
| Δρ_max_, Δρ_min_ (e Å^−3^) | 0.59, −0.54 |

The computer programs utilized in the study include CrysAlis PRO 1.171.38.46 (Rigaku OD, 2015), SHELXT, SHELXL (both developed by Sheldrick in 2015), and Olex2 (Dolomanov et al., 2009).

**Spectroscopic characterization**

Compound 6, which is an iminothiazoline derivative synthesized by following the aforementioned chemical reactions, was subjected to various characterization techniques. The Fourier Transform Infrared (FT-IR) spectrum of compound 6 displayed the presence of different types of bands. The C-H aromatic absorption bands were observed in the range of 3059-3115 cm-1, while the C-H thiazoline ring appeared at 2957 cm-1. The C-H alkyl groups gave rise to signals at 2858 and 2928 cm-1, and the C=O bond stretching vibration was observed at 1738 cm-1. In the proton nuclear magnetic resonance (1H NMR) spectrum of the compound, three distinctive signals were observed. The aromatic protons of the quinolinyl and phenyl rings appeared in the range of δ 8.95-7.28 ppm. The singlet of the proton located at the thiazoline ring was observed at δ 6.48 ppm, and the alkyl protons showed prominent signals in the range of δ 2.40-0.79 ppm. The carbon-13 nuclear magnetic resonance (13C NMR) spectrum displayed signals for carbonyl carbons at δ 174.7 and 173.6 ppm, while the signal for imine carbon of the thiazoline ring appeared at δ 170.8 ppm. The carbon located at the 5-position of the thiazoline ring gave rise to a signal at δ 104.5 ppm, and the signals for aromatic carbons of quinolinyl and phenyl rings appeared in the range of δ 149.6-126.4 ppm, while aliphatic carbons were located in the range of δ 29.1-13.6 ppm. These characteristic signals confirmed the successful formation of the desired iminothiazoline analogue. Further characterization of the synthesized compound was carried out using High-Performance Liquid Chromatography-Mass Spectroscopy (HPLC-MS) and X-Ray Diffraction (XRD) analysis.

**Elastase Inhibition Assay**

A 0.2 M Tris-HCl buffer with a pH of 8.0 was used to prepare a 0.8 mM solution of N-succinyl-Ala-Ala-Ala-p-nitroanilide. Next, 10 µL of the test sample was mixed with 130 µL of this buffer in a 96-well microplate. To evaluate the inhibition of elastase activity, we employed a modified version of a previously described method [1, 2], utilizing Elastase from the porcine pancreas. In this study, the release of p-nitroaniline from the substrate N-succinyl-Ala-Ala-Ala-p-nitroanilide by elastase was measured to determine the level of inhibition. Firstly, a 0.8 mM solution of N-succinyl-Ala-Ala-Ala-p-nitroanilide was prepared in 0.2 M Tris-HCl buffer (pH 8.0), and 130 µL of this buffer was mixed with 10 µL of the test sample in a 96-well microplate. The microplate was then pre-incubated for 10 minutes at 25ºC, followed by the addition of elastase (0.0375 Unit/mL) stock solution (10 µL). The microplate was maintained at 25ºC for 30 minutes, and the absorbance was read at 410 nm using a microplate reader (SpectraMax ABS, USA). To calculate the IC50 values, nonlinear regression was applied using GraphPad Prism 5.0 (GraphPad, San Diego, CA USA), and all experiments were conducted in triplicate. The inhibitory activities of elastase were then determined using the formula:

Elastase inhibition activity $\text{(\%) = (}\text{OD}\text{control}\text{ – }\text{OD}\text{sample}\text{ × 100) / }\text{OD}\text{control}$

The optical densities in the absence and presence of a sample were denoted as ODcontrol and ODsample, respectively. As a standard inhibitor for elastase, oleanolic acid was employed.

1. Ilyas, S., et al., *Preparation, structure determination, and in silico and in vitro Elastase inhibitory properties of substituted N-([1, 1′-Biphenyl]-2-ylcarbamothioyl)-Aryl/Alkyl benzamide Derivatives.* Journal of Molecular Structure, 2021. **1245**: p. 130993.

2. Ujan, R., et al., *Benzimidazole tethered thioureas as a new entry to elastase inhibition and free radical scavenging: Synthesis, molecular docking, and enzyme inhibitory kinetics.* Journal of Heterocyclic Chemistry, 2021. **58**(10): p. 1929-1935.
